# Chronic Lymphocytic Leukemia Involving the Breast Parenchyma, Mimicker of Invasive Breast Cancer: Differentiation on Breast MRI

**DOI:** 10.1155/2013/603614

**Published:** 2013-09-18

**Authors:** Vandana Dialani, Kalpana Mani, Nicole B. Johnson

**Affiliations:** ^1^Department of Radiology, Beth Israel Deaconess Medical Center, 330 Brookline Avenue, Boston, MA 02215, USA; ^2^Jefferson Radiology, Hartford, CT 06106, USA; ^3^Department of Pathology, Beth Israel Deaconess Medical Center, 330 Brookline Avenue, Boston, MA 02215, USA

## Abstract

Leukemic involvement of the breast is rare, particularly involvement by chronic lymphocytic leukemia (CLL). While concurrent invasive ductal carcinoma and CLL manifesting as a collision tumor in the breast is extremely rare, this association (CLL and carcinoma) has been described in other organs. We report here a case of a 58-year-old woman with concurrent invasive ductal carcinoma and CLL and describe the imaging features of CLL, particularly the differentiation on MRI.

## 1. Case History

A 58-year-old woman presented for screening breast MRI due to a high-risk history, specifically infiltrating breast carcinoma with ductal and lobular features involving the right breast in 1999, status after lumpectomy, tamoxifen therapy, and radiation therapy, without evidence of recurrence. Her medical history was also notable for chronic lymphocytic leukemia (CLL) diagnosed in 1998.

**Figure 1:**
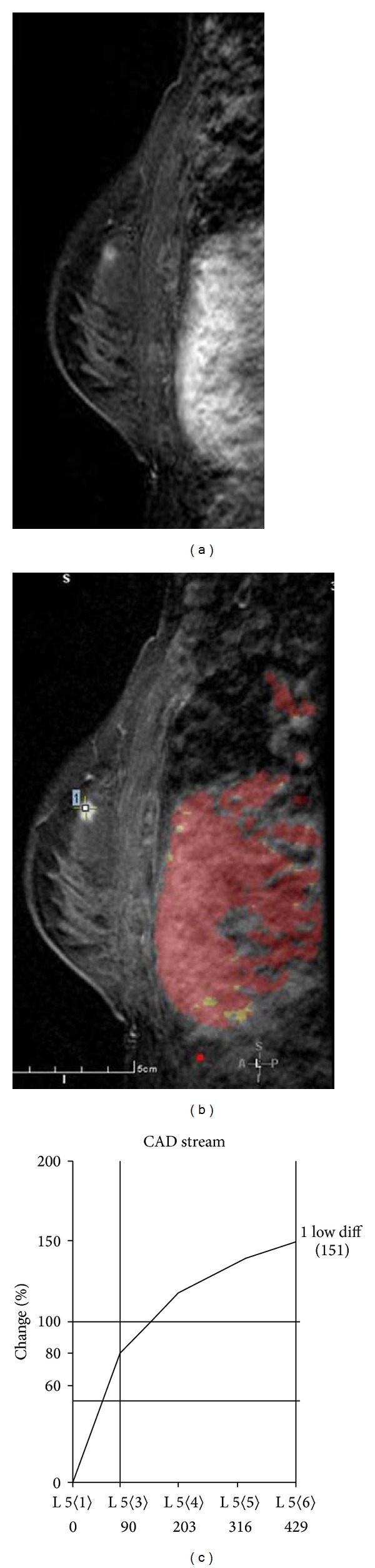
(a) A 6 mm new enhancing focus in the upper inner breast with slightly spiculated margins. (b) CAD image demonstrates that there is not enough enhancement to meet “threshold” value. (c) Graphical evaluation of enhancement demonstrates progressive type kinetics.

**Figure 2:**
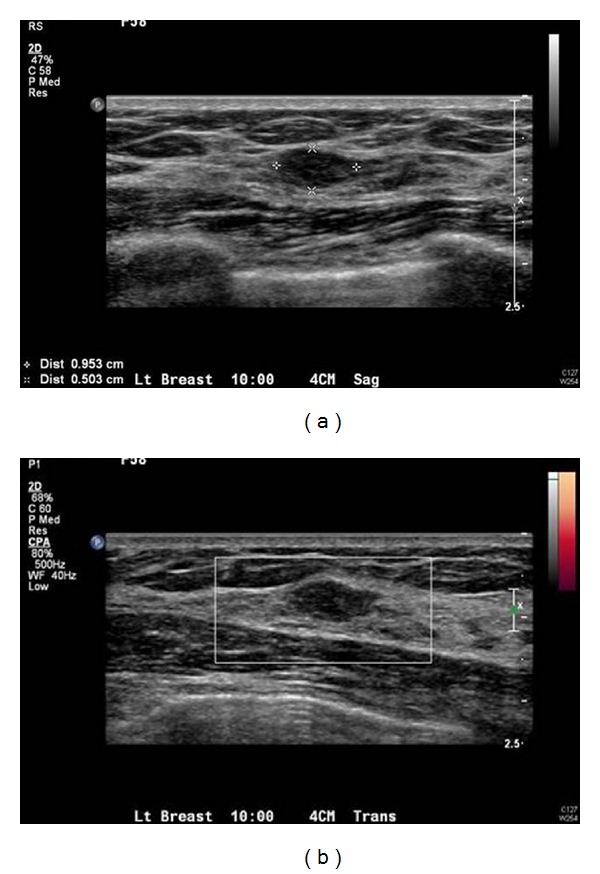
(a) Ultrasound evaluation in the upper inner breast demonstrates an oval, hypoechoic lesion, corresponding to the lesion seen on MRI (though slightly larger in dimension) with minimally irregular margins. (b) No increased vascularity.

**Figure 3:**
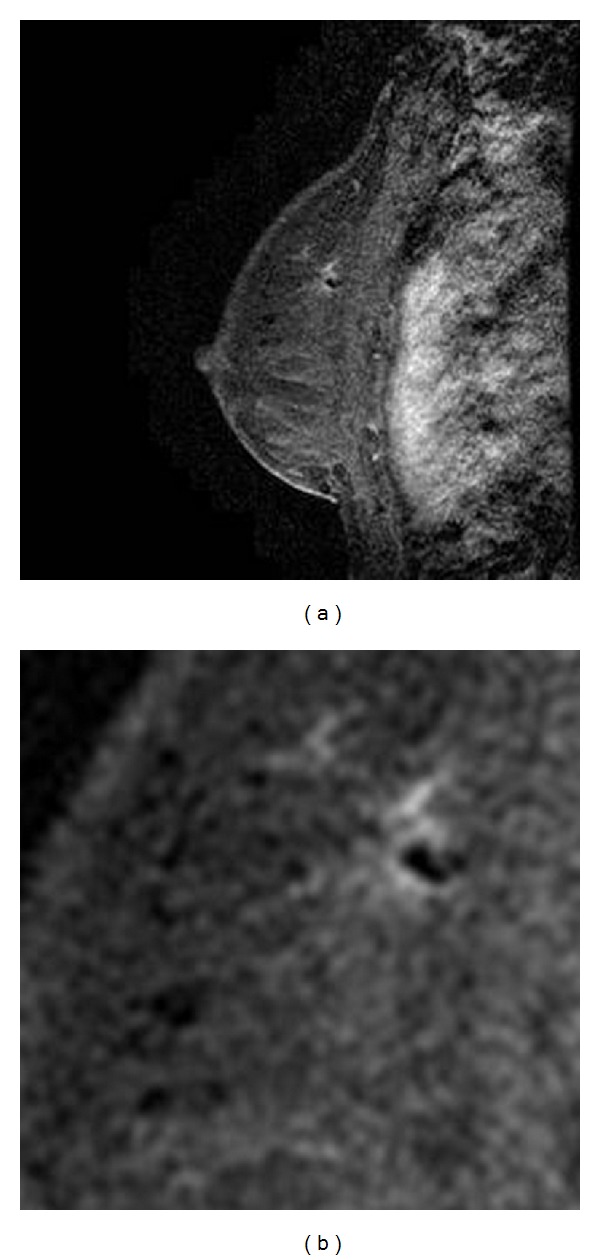
(a) Postclip placement MRI demonstrates a signal void (clip) within the enhancing nodule, confirming that the appropriate lesion was biopsied under ultrasound. (b) Coned down, magnified view of the same area.

Breast MRI performed on a 1.5T (GE Healthcare, Milwaukee, WI, USA), showed a new 6 mm lesion with slightly irregular margins in the upper inner quadrant of the left breast. The lesion did not meet threshold criteria for enhancement (100% increase in signal within 90 seconds), and the curve demonstrated slow progressive uptake of contrast within the lesion (Figures 1(a)–1(c)). Given its appearance and the patient's history of contralateral breast carcinoma, the enhancing lesion was categorized as a BIRADS 4B and biopsy was recommended. The lesion was mammographically occult. Focused ultrasound of the left breast demonstrated a hypoechoic, ovoid nodule at 10 o'clock, 4 cm from the nipple, oriented along the tissue planes, with slightly irregular margins. There was no abnormal vascularity (Figures 2(a) and 2(b)). A core biopsy under ultrasound guidance was performed, and an INRAD titanium clip was placed at the site of biopsy. An MRI of the breast was performed immediately after procedure and confirmed the clip placement within the enhancing lesion (Figures 3(a) and 3(b)).

**Figure 4:**
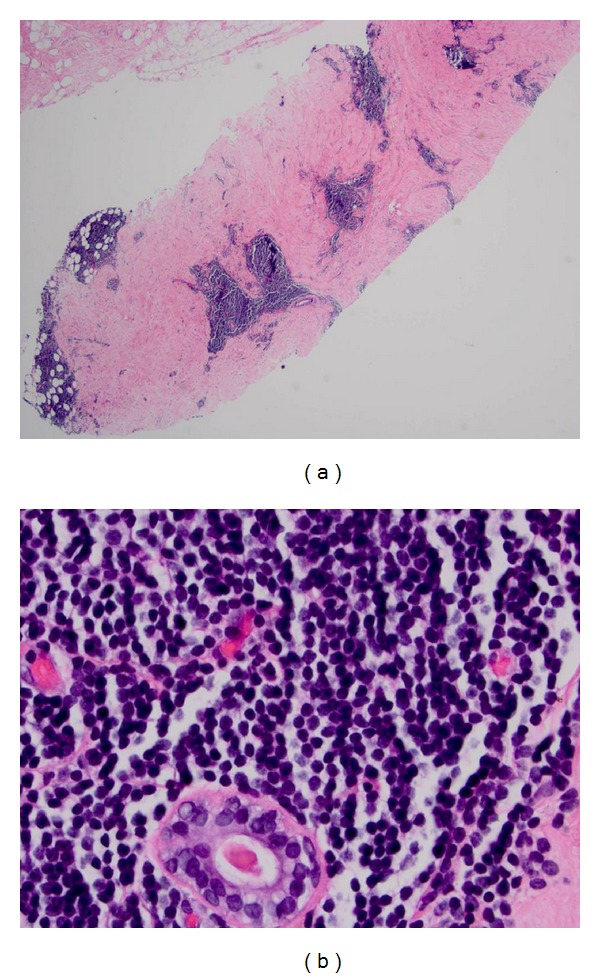
Chronic lymphocytic leukemia involving the breast. (a) Lymphocytic infiltrate is seen involving the stroma and adipose tissue (core biopsy, hematoxylin & eosin stain, 40x magnification). (b) Atypical small round monomorphic lymphocytes are present surrounding a benign duct (core biopsy, hematoxylin & eosin stain, 400x magnification).

Histologic examination of the core biopsy revealed multiple patchy foci of atypical lymphocytic infiltrates within the stroma and adipose tissue consisting of small mature monomorphic lymphocytes (Figures 4(a) and 4(b)). Immunohistochemistry displayed positive staining of these lymphocytes with CD20 and CD5. These cells were not reactive with CD3, CD23, or cyclin D1. These findings are consistent with involvement by the patient's known CLL. 

**Figure 5:**
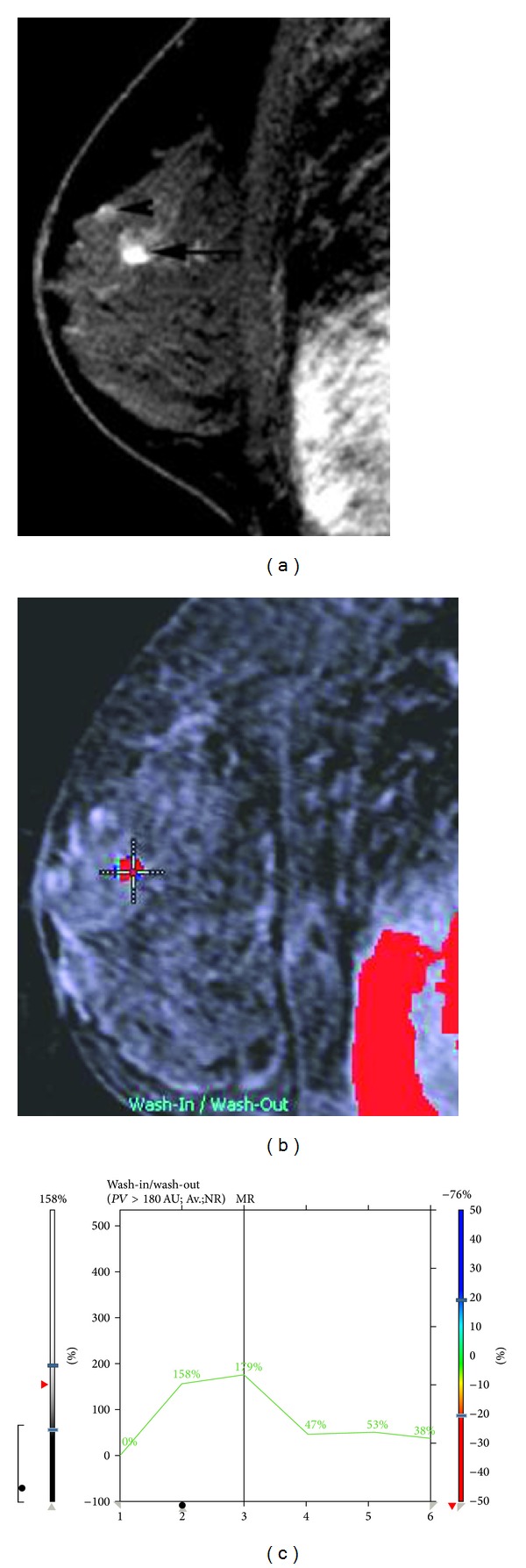
Eighteen months later, in the left breast a new distinct irregular 8 mm lesion (black arrow) and a 4 mm focus (arrowhead) were noted. The 8 mm irregular mass has wash-out kinetics (b), (c) for which MR-guided biopsy was recommended; the 4 mm focus does not meet threshold criteria for enhancement. Pathology of the suspicious lesion based on morphology and kinetics was invasive ductal carcinoma on histology. The anterior focus was involvement by CLL.

**Figure 6:**
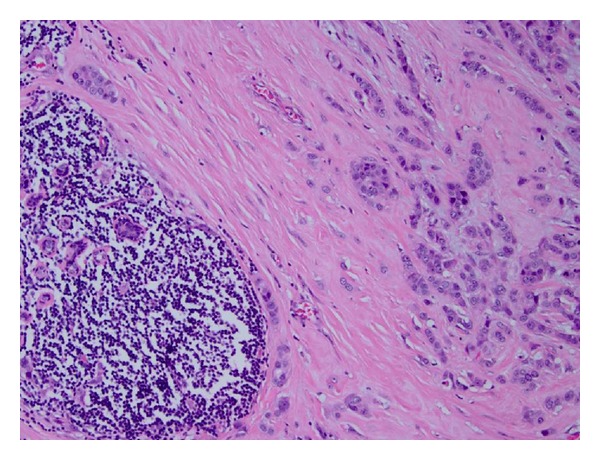
Infiltrating carcinoma cells, seen on the right side of the image, are present adjacent to an atypical lymphoid infiltrate consistent with chronic lymphocytic leukemia, present on the left side of the image (mastectomy specimen, hematoxylin & eosin stain, 100x magnification).

On further workup eighteen months later, in the left breast a new distinct irregular 8 mm lesion with early wash-in-wash-out kinetics was detected by MRI for which MR-guided biopsy was recommended and in addition adjacent 4 mm focus with benign kinetics, not meeting threshold criteria for enhancement, immediately adjacent to each other were noted (Figure 5). Core biopsy showed an 8 mm focus of invasive ductal carcinoma with adjacent foci of atypical lymphoid infiltrates, consistent with CLL, in the left breast parenchyma (Figure 6). The patient subsequently had bilateral mastectomies and sentinel lymph node biopsy on the left. Infiltration by CLL of the right breast and lymph nodes was also identified.

## 2. Discussion

Leukemic involvement of the breast is extremely rare, particularly involvement by CLL [1, 2]. Though rare, one of the more familiar leukemias which involve the breast is acute myelogenous leukemia, which can present with granulocytic sarcomas (chloromas) [3]. More rarely seen is acute lymphocytic leukemia, which can present as a diffuse bilateral breast process, an ill-defined mass, lymphadenopathy, or with a normal mammogram [4]. 

CLL can involve the breasts diffusely and bilaterally [5], include involvement of the overlying skin [1], present as an irregular breast nodule, be associated with microcalcifications [5], or be mammographically occult. In addition, CLL has been found synchronously in axillary lymph nodes ipsilateral to invasive breast cancer [6].

CLL manifesting in a collision tumor of the breast containing both invasive ductal carcinoma and foci of CLL is extremely rare [7, 8]. A postulated mechanism is that underlying etiology predisposes both tumor types (mutation of ATM gene or infection by Epstein-Barr virus) or that the CLL may express a B-cell receptor with affinity for an undefined breast cancer antigen [8]. A few reports describe other collision tumors in the breast such as association of invasive ductal carcinoma and mucosa-associated lymphoid (MALT) lymphoma [9, 10]. Etkind et al. have postulated a possible involvement of the presence of MMTV-like (mouse mammary tumor virus-like) envelope gene in patients with both invasive ductal carcinoma and non-Hodgkin's cell lymphomas [11]. 

CLL (chronic lymphocytic leukemia) and SLL (small lymphocytic lymphoma) are diagnosed primarily by morphology and secondarily by ancillary tools such as flow cytometry and immunohistochemistry. Confirmatory markers by flow cytometry include CD20 (dim), CD19, CD5 (aberrant coexpression), CD23, and light chain (dim). Immunohistochemistry performed in the tissue sections includes CD20 (dim), CD5 (dim), and CD23 [12]. Once a diagnosis of CLL/SLL is made, then additional prognostic markers include a FISH panel (including 13q [13]), IgVH, ZAP70 [14] is usually done on a case-by-case basis and the Rai-Binet score.

While MR imaging characteristics of breast primary and secondary lymphomas have been noted in the literature [9], to our knowledge, this report is the first in which CLL infiltration of the breast has been evaluated by MRI. The MRI findings were concordant with an indolent process; the enhancement within the small lesion did not meet threshold criteria and demonstrated progressive rather than wash-out kinetics, as against the invasive carcinoma which showed distinct early wash-in and wash-out kinetics. In addition, the lesion was neither palpable nor painful, and there were no associated overlying skin findings nor axillary lymphadenopathy. On ultrasound, the lesion was indeterminate with some irregularity of margins but respecting the tissue planes. 

## 3. Conclusion

 It is important to consider involvement of the breast by hematologic malignancies specifically in patients with known systemic involvement, when MRI shows indolent findings as described, so that appropriate followup can be provided for the patient.
